# Novel Use of Chicory for the Treatment of Hiccups in Liver Obstruction: In Iranian Traditional Medicine

**DOI:** 10.5812/ircmj.6647

**Published:** 2013-11-05

**Authors:** Qadir Mohammadi, Mohamad Bagher Minae, Mohammad Hosein Somi, Mahmoud Mosaddegh, Mohammad Kamalinejad

**Affiliations:** 1 School of Iranian Traditional Medicine Shahid Beheshti University of Medical Sciences, Tehran, IR Iran; 2 Research Institute for Islamic and Complementary Medicine, Tehran University of Medical Sciences, Tehran, IR Iran; 3 Tabriz Liver and Gastrointestinal disease research center, Tabriz, IR Iran; 4 Department of Pharmacognosy, School of Pharmacy, Shahid Beheshti University of Medical Sciences, Tehran, IR Iran

Dear Editor,

A hiccup is the result of an involuntary, intermittent spasmodic contraction of the diaphragm and the inspiratory intercostal muscles. Based on Iranian Traditional Medicine, hiccups are stomach diseases and movements in the stomach with the combination of spasmodic contraction followed by a stretching distention (1). Although hiccups may be a temporary action which need no treatment, sometimes a refractory hiccup would be disturbing to a normal life. According to Avicenna, a hiccup may occur due to different causes, one of which occurs because of liver obstruction. We experienced the successful use of Chicory to cure two cases of hiccups caused by liver obstruction and inflammation in digestion Clinic of Shahid Beheshti Medical Faculty. The first case was a 78 year old male with two years of refractory and chronic hiccup, who was under a common drug treatment. Due to his refusal in using the drugs because of their side effects, his hiccups were continuous and loud enough to disturb his sleep, eating routines. It also was so severe that made him depressed. Other symptoms included pains and heaviness in RUQ. These two symptoms based on Avicenna are Pathgnomonic of liver obstruction. This was resolved by one dose of 10% syrup of Chicory, which was orally taken. It was used for three weeks before breakfast. In the middle of the second week, his problem was slighter so that it was completely gone by the end of third week. The subject was under control for six months. The problem appeared again at the end of the third month, so we started the same treatment. By the end of the sixth months, he never complained. The other patient was a 54 year old male with an intermittent hiccup, which occurred almost every day, worsening lately. He had been under medical treatment; however, his hiccups were not completely cured. The same treatment was prescribed to him in three weeks and after the 2nd week, the hiccup was resolved and there was seen no relapse within six months of control.

In the Allopathic medicine hiccup etiology is not pretty clear. As far as it is known hiccups are caused by various factors such as neurologic or non-neurologic agents, cerebral tumors, prostate cancer, abdomen surgery, MI, hepatitis, gastritis, duodenitis, esophageal reflux, peptic ulcer, and esophagitis (2-4). To cure a hiccup, in the Allopathic medicine metoclopramide, chlorpromazine, baclofen, gabapentin, Phernic nerve neurolysis, and nerve blocks are used (2, 4). The best result was obtained by using Gabapentin in acute cases. In Traditional medicine, which goes back to Hippocrates, BC ^460^ hiccups are sometimes defined as a symptom of prognosis and it has been also mentioned that if hiccups are accompanied with red eyes, it indicates bad prognosis which can be indicative of encephalitis, gastritis, and neuritis attaching stomach and brain (1, 5).

In Avicenna's view, hiccups are known as stomach diseases. He relates it to the cardia of stomach. Hiccups sometimes happen due to the interaction between stomach and diseases in other organs such as hepatitis and liver obstruction. In this study we investigated those hiccups caused by liver disease (5).

Chicory belongs to the Composite family with the scientific name of Chicorium Intybus L. which is used as Diuretic, appetizer, and cholagogue (6-8). In ITM (Iranian Traditional Medicine), Chicory is known as Handba and is an Opener that is the literal translation for MOFATTEH. It is bile and blood intensity and Heddat (Acuity) reliever, and its root is deeply opener and MOLATTEF (to dilute dense humor with moderate heat) of humors (3). It is also useful for refining blood and swelling organs. Its usage dose is from 4 to 12 grams (9). Considering the weak effects of chemical drugs on treatment of refractory hiccups and their side effects, Chicory which was also proved useful in this study with no recorded side effect, (10) is recommended as an appropriate treatment for chronic hiccups caused by liver disease.
